# TransfersomILs: From Ionic Liquids to a New Class of Nanovesicular Systems

**DOI:** 10.3390/nano12010007

**Published:** 2021-12-21

**Authors:** Ana Júlio, João Guilherme Costa, Catarina Pereira-Leite, Tânia Santos de Almeida

**Affiliations:** 1 CBIOS—Universidade Lusófona’s Research Center for Biosciences & Health Technologies, Campo Grande 376, 1749-024 Lisboa, Portugal; ana.julio@ulusofona.pt (A.J.); jgcosta@ulusofona.pt (J.G.C.); catarina.leite@ulusofona.pt (C.P.-L.); 2 Department of Biomedical Sciences, University of Alcalá, Ctra. Madrid-Barcelona Km. 33.600, Alcalá de Henares, 28871 Madrid, Spain; 3 LAQV, REQUIMTE, Departamento de Ciências Químicas, Faculdade de Farmácia, Universidade do Porto, 4050-313 Porto, Portugal; 4 Centro de Química Estrutural, Faculdade de Ciências, Universidade de Lisboa, Campo Grande, 1749-016 Lisboa, Portugal

**Keywords:** ionic liquids, nanosystems, rutin, Box–Behnken factorial design, TransfersomILs, cutaneous delivery

## Abstract

Ionic liquids (ILs) have increasingly been studied as key materials to upgrade the performance of many pharmaceutical formulations. In controlled delivery systems, ILs have improved multiple physicochemical properties, showing the relevance of continuing to study their incorporation into these formulations. Transfersomes are biocompatible nanovesicular systems, quite useful in controlled delivery. They have promising characteristics, such as elasticity and deformability, making them suitable for cutaneous delivery. Nonetheless, their overall properties and performance may still be improved. Herein, new TransfersomILs systems to load rutin were developed and the physicochemical properties of the formulations were assessed. These systems were prepared based on an optimized formulation obtained from a Box–Behnken factorial design (BBD). The impact of imidazole-based ILs, cholinium-based ILs, and their combinations on the cell viability of HaCaT cells and on the solubility of rutin was initially assessed. The newly developed TransfersomILs containing rutin presented a smaller size and, in general, a higher association efficiency, loading capacity, and total amount of drug release compared to the formulation without IL. The ILs also promoted the colloidal stability of the vesicles, upgrading storage stability. Thus, ILs were a bridge to develop new TransfersomILs systems with an overall improved performance.

## 1. Introduction

Over the years, ionic liquids (ILs) have generated a growing interest concerning their applicability in the pharmaceutical field, particularly due to the fact of their multifunctionality [1,2,3,4,5]. In fact, these compounds have remarkable properties, such as a high thermal and chemical stability [3], non-flammability [3], wide liquid range [5], and remarkable dissolution properties [5,6], all of which are characteristics that distinguish them from other organic solvents, namely, for applicability in the pharmaceutical area [3,5,7]. Consequently, ILs have been used as solvents [2,8], as excipients in various formulations [2,4,9,10,11,12,13,14], as solubility [2,10,11,13,14,15,16] and permeability [10,13,17,18,19] promoters, as surface active ILs [20,21,22,23], to improve the functionality of biomolecules such as proteins and enzymes [24,25,26,27], and also in drug delivery [4,15,16,17,28,29,30,31], amongst others [7]. Some of them have also been called green solvents, due to the fact of their low toxicity (compared to other ILs), making them key materials, particularly in drug delivery strategies [2,3,4,5,29,32,33]. Thus, ILs present great potential and have gained relevance in the development of innovative drug delivery systems. In fact, ILs have been used as part of various types of delivery systems to improve their performance and some studies have shown that their inclusion may even lead to multiple upgrades in developed systems and not only to a single functionalization [2,3,4,29,32]. For instance, ILs may not only be valuable to enhance the incorporation of poorly soluble drugs into topical formulations, such as oil-in-water (O/W) emulsions, but they may also increase the viscosity and the stability of these thermodynamically unstable systems [2].

ILs may also be key to improve multiple characteristics of controlled drug delivery systems such as lipidic implants [4] and IL–nanoparticles hybrid systems [16,28,32,34,35,36]. In fact, since controlled delivery systems are quite relevant for long-term therapies, investing in the study of new materials that may lead to more effective formulations of this type, remains essential. 

Transfersomes are among this type of formulations. They are nanovesicular systems with a remarkable skin permeation capability due to the incorporation of edge activators (EAs) within the phospholipid bilayer that surrounds an aqueous compartment [37]. EAs act as bilayer-destabilizing agents, thereby pronouncedly increasing the elasticity and deformability of the nanovesicles, allowing the nanocarriers to transport therapeutic agents to deeper layers of the skin [38]. The most used EAs in transfersomal formulations include non-ionic surfactants and bile acids, as recently reviewed [37,38]. It is noteworthy that it is crucial to optimize the phospholipid–EAs ratio to ensure that the nanovesicles display the penetration-enhancing feature and maintain their structural integrity. In this sense, quality-by-design strategies are an asset to direct the product development towards the predefined objectives, while reducing the manufacturing costs as well as time and materials waste [37]. This type of strategy is particularly relevant when designing transfersomes, as the raw materials used, namely, phospholipids, are quite expensive. This aspect justifies the use of various quality-by-design strategies, such as Box–Behnken factorial design and Plackett–Burman design, in recent publications concerning transfersomes development [39,40,41]. Another well-known drawback of transfersomal formulations is their poor storage stability, due to the fact of their tendency to aggregate, oxidize, and their high water content, the reason why they are usually stored at low temperatures [38]. To overcome this limitation, it would be interesting to include novel excipients in these formulations with antioxidant or antimicrobial properties. Despite these two less attractive points, the potential of transfersomes to load both hydrophilic and hydrophobic compounds and to provide a sustainable delivery of these agents across the skin justifies the continuous interest of the scientific community in translating these nanovesicular systems into clinical applications.

Rutin, a hydrophobic polyphenolic bioflavonoid found in many natural sources, has shown pharmaceutical interest. Several studies have been describing its potential as anti-inflammatory, antioxidant, and anticancer [42,43,44]. In this context, rutin has been included in topical formulations for photoprotective and anti-aging purposes [45,46] and for treating atopic and allergic contact dermatitis [47]. The anti-melanoma activity of rutin has also been demonstrated in cell-based studies [48,49]. Thus, the development of rutin-loaded topical formulations may be valuable for cosmetic and therapeutic applications. Nonetheless, this compound has a high molecular weight that impairs its penetration through the skin, with this being the reason why various nanosystems have been developed to load rutin [16,28,32,50,51,52,53]. Consequently, improving the incorporation of this compound into transfersomes could be a profitable strategy to further enhance the cutaneous permeability of rutin. 

Hence, we aimed to develop new TransfersomILs (transfersomes containing ILs) to load rutin for cutaneous applications and to evaluate the impact of the studied ILs and IL:IL combinations on the physicochemical properties of the prepared formulations. The ILs were chosen since they have previously shown promising features that justify further investigation [2]. The solubility of rutin in aqueous solutions containing IL:IL combinations of imidazole-based ILs and choline-based ILs was determined as well as their impact on the cell viability of human keratinocytes. Secondly, the developed transfersomal system was optimized using a Box–Behnken factorial design (BBD). Then, the imidazole-based ILs, the choline-based ILs as well as their combinations were incorporated into the optimized formulation. The physicochemical properties and the performance in terms of in vitro drug release and storage stability of the developed systems were assessed. This allowed to evaluate if the TransfersomILs could represent an innovative and advantageous strategy in skin drug delivery.

## 2. Materials and Methods

### 2.1. Materials and Reagents

For the synthesis of the ILs, choline hydroxide in methanol [Cho][OH]/MeOH 45%, methanol, Amberlite^®^ IRA-400 chloride form, 1-bromoethane, and glycine from Sigma–Aldrich (Saint Louis, MO, USA) were used as well as 1-methylimidazole and acetonitrile from VWR (Fontenay-sous-Bois, France) and sodium hydroxide (NaOH) from PanReact AppliChem (Barcelona, Spain). Rutin was obtained from Fragron (São Paulo, Brazil).

Regarding the cell viability studies, trypsin, penicillin–streptomycin solution, fetal bovine serum, dimethyl sulfoxide (DMSO), and thiazolyl blue tetrazolium bromide (MTT) were acquired from Sigma–Aldrich (Saint Louis, MO, USA) and Dulbecco’s modified Eagle’s medium (DMEM) was purchased by Biowest (Nuaillé, France).

For the preparation of transfersomes, methanol was from Carlo Erba Reagenti SpA (Rodano, Italy), chloroform from Sigma–Aldrich Chemie Gmbh (Munich, Germany), Tween^®^ 80 from Sigma–Aldrich (Saint Louis, MO, USA), and soy phosphatidylcholine from Alfa Aesar (Kandel, Germany). For the release study, phosphate-buffered saline (PBS, pH 7.4) was prepared as previously described [54].

### 2.2. Synthesis of ILs

In this work, the studied ILs were 1-ethyl-3-methylimidazolium bromide [Emim][Br], 2-hydroxyethyl-trimethylammonium glycinate [Cho][Gly], and 1-ethyl-3-methylimidazolium glycinate [Emim][Gly], which were synthesized and characterized within the scope of another recently published study from our group [2]. Briefly, for [Emim][Br] 135 mmol of 1-bromoethane was added, drop by drop, to 45 mmol of 1-mehtylimidazol. This blend remained overnight under stirring at room temperature and was then evaporated under vacuum. For [Cho][Gly], 57.79 mmol of glycine, solubilized in water, was added to 57.76 mmol of the evaporated choline hydroxide. This mixture was stirred overnight, the solvent was evaporated and then an acetonitrile:methanol (9:1) mixture was added. The blend was centrifuged (at 1500 rpm for 30 min) and after filtration, the solvent was evaporated. Concerning the [Emim][Gly], first the [Emim][OH] was prepared by ion exchange chromatography using 10.47 mmol of [Emim][Br] as described in the literature [2]. The obtained combined fractions were added drop by drop to an aqueous solution of glycine in molar excess. The mixture was stirred overnight in an ice bath, then evaporated under vacuum and the unreacted amino acid precipitated with acetonitrile was removed by centrifugation at 450× *g* followed by filtration. The solvent was removed by evaporation.

After being synthesized, all ILs were stored under moisture-free conditions.

### 2.3. Cell Viability Study

The cell viability was performed in human keratinocytes (HaCaT). Cells were cultured in DMEM supplemented with 10% fetal bovine serum and 1% penicillin–streptomycin. HaCaT cells were maintained at 37 °C under a humidified air atmosphere containing 5% of CO_2_ in air.

Approximately 6 × 10^3^ cells were seeded per well in 96-well plates in 200 µL of culture medium and incubated for 24 h. Afterwards, HaCaT cells were exposed to the aqueous solutions containing each IL (0.1% or 0.2% *v*/*v*) and the IL combinations (99.8:0.1:0.1% *v*/*v*), for a 24 h period. The cell viability was assessed by MTT reduction assay, according to previously described protocols [55,56]. Absorbance values for the untreated control cells correspond to 100% of cell viability. For this assay, three independent experiments were performed, and four replicate cultures were used in each experiment.

### 2.4. Solubility Studies

The solubility studies were performed as previously described in the literature [2]. Briefly, rutin saturated solutions were prepared in water, water:IL mixtures (99.9:0.1 or 99.8:0.2% *w*/*w*) or water:IL:IL combinations (99.8:0.1:0.1% *w*/*w*). The studied ILs were [Emim][Br], [Cho][Gly], and [Emim][Gly] and their combinations [Cho][Gly]:[Emim][Br] and [Cho][Gly]:[Emim][Gly].

All the solutions were prepared in triplicate and stirred on a horizontal shaker (IKA VIBRAX VXR^®^, LTF Labortechnik GmbH & Co., Bodensee, Germany) for 72 h at 25 ± 2 °C. Then, to determine drug solubility, the samples were filtered and analyzed using an UV-Visible spectrophotometer (Evolution^®^ 300, Thermo Scientific, Hertfordshire, UK) at 353 nm (the maximum absorption wavelength for rutin in water).

### 2.5. Transfersomes Preparation

The transfersomes were produced by the thin-film hydration method followed by sonication as previously described with some modifications [39]. Briefly, soy phosphatidylcholine and Tween^®^ 80 were dissolved in chloroform:methanol (3:1, *v*/*v*). The mixture was placed in a rotary evaporator at 40 °C for 45 min to evaporate the organic solvents and remained under vacuum to remove their traces, forming the EA:lipid films. Then, they were hydrated with a rutin solution in water, in water:IL mixtures, or in water:IL:IL combinations, vigorously vortexed, and sonicated at 50% amplitude using a Q125 Sonicator from QSonica Sonicators (Newtown, CT, USA). Finally, the produced transfersomes were allowed to equilibrate at 200 rpm/min for 30 min in a horizontal shaker (IKA VIBRAX VXR^®^, LTF Labortechnik GmbH & Co., Bodensee, Germany). 

### 2.6. Box–Behnken Factorial Design

The optimization of the rutin-loaded transfersomes was based on a 15-run, 3-factor, 3-level Box–Behnken factorial design (BBD). The independent variables, also called factors, were lipid concentration (X_1_), the EA:lipid ratio (X_2_), and the sonication time (X_3_). Three levels of each factor were tested, as detailed in Table 1 and they were selected according to our preliminary results and literature research [39,40,41]. The responses or dependent variables were the hydrodynamic diameter, D_h_ (Y_1_), the polydispersity index, PDI (Y_2_), the association efficiency, AE (Y_3_), and the loading capacity, LC (Y_4_). The corresponding desirable criteria were defined for each dependent variable (Table 1).

The obtained results were analyzed in STATISTICA^®^ software (Statsoft, Inc., Tulsa, OK, USA) to predict the optimum levels of factors to produce the optimized formulation. To validate the experimental design, three replicates of the optimized formulation were produced and characterized to compare the experimental responses with the theoretical values predicted by the BBD.

### 2.7. Physicochemical Characterization of the Transfersomes

The produced formulations were characterized by the analysis of the hydrodynamic diameter (D_h_) and the polydispersity index (PDI) using Delsa™ Nano C from Beckman Coulter, Inc. (Brea, CA, USA), after their dilution (50×) with bidistilled water. The zeta potential (ZP) was also measured after diluting the sample 25× in bidistilled water, using a ZetaPALS/ZetaPotential Analyzer (Brookhaven Instruments, Holtsville, NY, USA). All samples were analyzed in triplicate at 23 ± 2 °C.

The AE and LC were also evaluated using an indirect method. After a dilution of 1:10 (*v*/*v*), 500 µL of each sample was placed in a VIVASPIN^®^ 500 centrifuge tube (Sartorius, Goettingen, Germany), prior to centrifugation at 12,000× *g* for 30 min in a Hermle Z 32 HK centrifuge, from Hermle LaborTechnik (Wehingen, Germany). The supernatant was then diluted in an ethanol:water mixture (8.5:1.5) and the non-loaded fraction of rutin was quantified using an UV–Visible spectrophotometer (Evolution^®^ 300, Thermo Scientific, Hertfordshire, England), at its maximum absorption wavelength (353 nm).

The percentage of AE (1) and LC (2) was calculated using the following equations:

(1) $$\%AE=\frac{Total\;\left(rutin\right)-Non-loaded\;\left(rutin\right)}{Total\;\left(rutin\right)}\times 100$$

(2) $$\%LC=\frac{Total\;\left(rutin\right)-Non-loaded\;\left(rutin\right)}{Total\;\left(lipid\right)}\times 100$$

### 2.8. In Vitro Release Studies

In vitro release studies were performed according to a dialysis bag diffusion method. The transfersomal formulations (1.5 mL) were incorporated in a dialysis bag (CelluSep^®^ H1 with a nominal molecular weight cutoff of 2000 Da from Uptima^®^ of Interchim, Montluçon, France). Then, to simulate the physiological conditions, the systems were placed in the PBS buffer at 37 ± 2 °C under stirring. An aliquot of the external medium was collected and immediately replaced by the same volume of PBS at various time intervals. Sink conditions were kept throughout the study. To quantify the released amount of rutin at each time point, UV–Visible spectrophotometry (Evolution^®^ 300, Thermo Scientific, Hertfordshire, UK) was used at the drug’s maximum absorption wavelength (353 nm). The release profiles were obtained by considering the cumulative amount of rutin released, in percentage, versus time. 

### 2.9. Preliminary Stability Studies

Immediately after preparation, all produced transfersomal formulations were stored in refrigerated conditions (5 ± 2 °C) for 90 days. To evaluate the storage stability, the transfersomes were submitted to D_h_ and PDI analyses after preparation and at days 15, 30, 45, 60, and 90 as described in Section 2.7.

### 2.10. Statistical Analysis

The results are expressed as the mean ± standard deviation (SD). After conducting normality and homogeneity tests, data from solubility studies were evaluated by one-way analysis of variance (ANOVA) followed by Tukey’s multiple comparison test. The following results from other studies were evaluated by two-way ANOVA, followed by Bonferroni post hoc test. The differences between individual means were significant at * *p* < 0.05, ** *p* < 0.01, and *** *p* < 0.001. The analyses were performed using the SPSS^®^ statistical package (version 25, SPSS Inc. Chicago, IL, USA).

## 3. Results and Discussion

**Figure 1:**
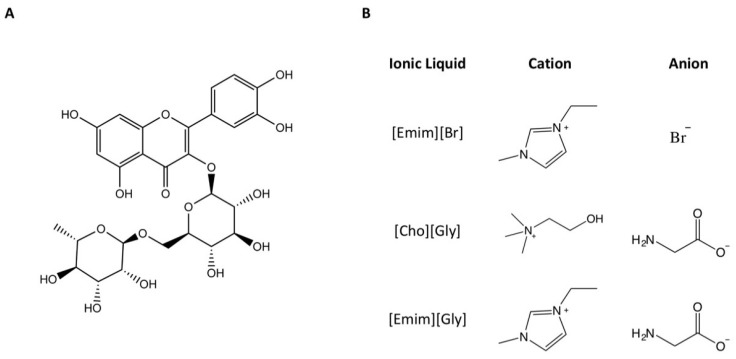
Chemical structures of rutin (**A**) and the studied ionic liquids (**B**).

Although transfersomes present many advantages, such as being biocompatible, suitable for delivering both lipophilic and hydrophilic drugs, and being deformable, these systems may still be improved, namely, in terms of their physicochemical properties and their storage stability. Hence, transfersomal formulations containing rutin (Figure 1A) were prepared in the absence and in the presence of ILs to evaluate the impact of these materials on the physicochemical properties of the vesicular systems. The studied ILs (Figure 1B) were 1-ethyl-3-methylimidazolium bromide [Emim][Br], (2-hydroxyethyl)trimethylammonium glycinate [Cho][Gly], 1-ethyl-3-methylimidazolium glycinate [Emim][Gly], and their combinations. These ILs were chosen because they have shown promising characteristics for use in topical drug delivery such as drug solubility and loading enhancement [2]. 

### 3.1. Viability and Solubility Studies

It is known that [Emim][Br], [Cho][Gly], and [Emim][Gly] improve the stability of O/W emulsions containing rutin, and that [Cho][Gly] and [Emim][Gly] improve the aqueous solubility of this and other phenolic compounds [2]. Thus, incorporating these ILs alone and their combinations into transfersomes containing rutin may be key, and to the best of our knowledge, this is the first time that this approach has been evaluated. 

To achieve this, the ILs should be included at non-toxic concentrations and, thus, initially, the MTT assay was used to evaluate the impact of each IL and of each combination of ILs on the viability of HaCaT cells (0–0.2% *v*/*v*; 24 h). The combinations of ILs studied were [Cho][Gly]:[Emim][Br] and [Cho][Gly]:[Emim][Gly] (both at 0.1:0.1% *v*/*v*). The impact of these combinations on HaCaT cells’ viability was studied herein for the first time. Our present results (Table 2) confirm the previously published data concerning the impact of the ILs alone in this cellular model [2] and reveal that the studied combinations of ILs also maintain the cell viability of HaCaT cells (above 90% for all ILs). Thus, this indicates that the evaluated concentrations may be safely incorporated into the transfersomal systems.

Following this, the impact of the combination of ILs on the aqueous solubility of rutin needed to be assessed. To understand this impact, the solubility of rutin was studied in several solutions. Namely, in water alone, in water:IL mixtures, and in the water:IL:IL combinations (Table 2). Our results showed that the solubility in water and in water:[Emim][Br], water:[Cho][Gly], and water:[Emim][Gly] are all in agreement with the literature [2]. Concerning the impact of the combinations of ILs on the drug solubility, our results unveiled for the first time that both [Cho][Gly]:[Emim][Br] and [Cho][Gly]:[Emim][Gly] combinations enhanced the solubility of rutin, although this enhancement was slightly lower when compared to [Cho][Gly] or [Emim][Gly] alone. The observed enhancement in drug aqueous solubility may be due to the hydrotropic character of the studied ILs, as it has been shown for other imidazolium- and cholinium-based ILs [20,57,58,59]. For instance, Sintra et al. [57] have shown that a mechanism based in drug-IL aggregation is behind the solubility enhancement observed for ibuprofen, another poorly water-soluble drug. Moreover, it has also been reported that ILs containing amino acids play a key role in hydrotropic solubilization [60]. Additionally, the results also showed that the [Cho][Gly]:[Emim][Gly] blend led to a higher increase, proving to be a more successful strategy to improve rutin’s solubility. This result is somewhat expected, since individually [Emim][Br] did not impact the solubility of rutin (Table 2). Consequently, a combination of ILs that includes [Emim][Br] could be expected to be less efficient in terms of solubility. Still, since imidazolium-based ILs are known to impact other properties, such as skin permeation [13,18,61], their inclusion (as well as the inclusion of different combinations of ILs) into the transfersomes may still be relevant to upgrade other functionalities, because different cations and anions may affect distinct properties. 

After studying the impact of the ILs and of their combinations on cell viability and on rutin’s solubility, we started by optimizing a transfersomal system to load rutin in the absence of ILs, using a Box–Behnken factorial design (BBD).

### 3.2. Optimization of Rutin-Loaded Transfersomes: Box–Behnken Design

The development of transfersomes to load rutin was based on a 15-run, 3-factor, 3-level BBD, meaning that we chose three levels for each selected factor, as described in Table 1, resulting in 15 formulations to be produced according to the STATISTICA^®^ software. After being prepared, each formulation was characterized in terms of the selected responses to be evaluated, namely, hydrodynamic diameter (D_h_), polydispersity index (PDI), association efficiency (AE), and loading capacity (LC) as described in the Supplementary Materials (Table S1). Overall, D_h_ varied between 104 and 116 nm and PDI between 0.25 and 0.28, suggesting that all 15 formulations displayed diameter (D_h_ < 300 nm) and size distribution homogeneity (PDI < 0.3), which are favorable properties to be successfully applied on the skin [37,38]. Moreover, AE and LC values were satisfactory, varying from 74% to 91% and from 0.21% to 0.44%, respectively. Considering the desirability criteria initially chosen for each response (Table 1), the obtained results indicate that the factors and respective levels initially selected were appropriate.

The regression analysis of the aforementioned results, considering a 95% confidence level, was based on the two-way interactions (linear × quadratic) model, since it was the fitting model yielding the highest *R*^2^ values for each evaluated response: 0.95565 for D_h_, 0.94318 for PDI, 0.99287 for AE, and 0.99915 for LC. These values indicate that the experimental and theoretical values were well correlated and confirm that a cubic regression model is needed to fit the experimental data to ultimately predict the most suitable factor levels for producing the optimized formulation. In order to evaluate the effects of each factor or of their linear or quadratic relationships on the obtained responses, coefficients and *p*-values were calculated from the regression analyses as detailed in the Supplementary Materials (Table S2). 

Regarding D_h_ and PDI, no significant linear or quadratic effects were observed for any factor, in agreement with the narrow interval of D_h_ and PDI values obtained with the 15 produced formulations. In contrast, both synergistic (i.e., positive coefficient) and antagonistic (i.e., negative coefficient) significant effects were observed for AE and LC. Concerning AE, a positive and linear effect of the lipid concentration was found, showing that the higher the lipid concentration used, the higher the AE of transfersomes. Moreover, a synergistic (i.e., linear and quadratic) effect of sonication time was also observed for AE, since increasing the sonication time also increased AE. In contrast, the interaction effects of lipid concentration and EA:lipid ratio (X_1_X_2_) as well as of lipid concentration and sonication time (X_1_X_3_) were antagonistic, showing that the increase of X_1_X_2_ or X_1_X_3_ values decreased the obtained values of AE. Regarding LC, an antagonistic, linear, and quadratic effect of lipid concentration was observed, meaning that LC increased by decreasing the lipid concentration. In contrast, the sonication time caused a synergistic effect in a linear and quadratic manner as reported for AE. Finally, it should be noted that only one interaction effect was statistically significant, namely, the linear relationship between lipid concentration and sonication time (X_1_X_3_), which caused an antagonistic effect.

**Figure 2:**
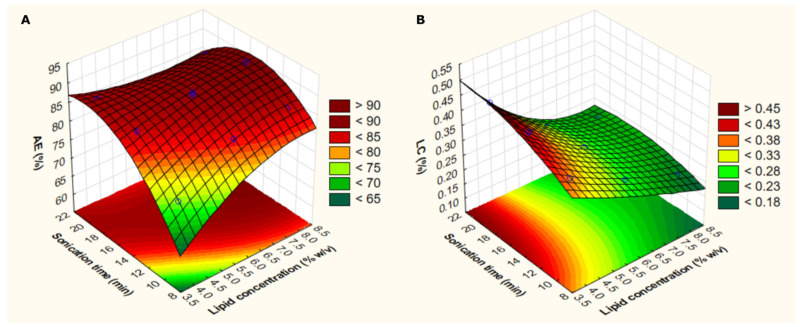
3D–response surface plots considering lipid concentration and sonication time according to the analyzed response (association efficiency: AE—(**A**); or loading capacity: LC—(**B**)). Light green indicates the lowest response level and dark red indicates the highest response level.

3D–response surface analyses (Figure 2) were determined to better show the statistically significant effects of two factors (i.e., lipid concentration and sonication time), while maintaining constant (at level 0) the EA:lipid ratio. It is noteworthy that AE values superior to 85% can be obtained when using lipid concentrations higher than 6.5% and sonication times higher than 13 min. In contrast, the highest LC values (>0.45%) can be obtained when using low lipid concentrations (<4%) and high sonication times (>16 min).

Considering the performed regression analyses and the desirability criteria initially selected (Table 1), the STATISTICA^®^ software was used to calculate the response desirability profile and to predict the most suitable levels of each factor to produce the optimized transfersomes (Table 3). To verify the validity of the prediction ability of the model, the optimized formulation was produced in triplicate, and it was characterized in terms of D_h_, PDI, AE, and LC. The obtained experimental values were further compared with the theoretical values predicted by the model as detailed in Table 3. As the experimental and theoretical data were similar, the implementation of this BBD was a valid quality-by-design approach to optimize rutin-loaded transfersomes.

By comparing the physicochemical properties of the optimized transfersomes obtained herein with other published studies concerning nanovesicular systems to load rutin [62,63,64,65,66], it is clear that this work is a remarkable breakthrough. This transfersomal formulation displayed low particle size combined with low PDI in contrast with other liposomal [62,63,65] or ethosomal [64] formulations, while maintaining the highest value reported for AE. Since it was already described the lower the vesicle size, the higher the skin penetration [66], these rutin-loaded transfersomes are expected to display enhanced skin permeation than the other nanovesicular formulations developed so far. Moreover, the presence of EA in the formulation may further increase the flexibility of the vesicles, contributing even more to the efficient delivery of rutin upon skin application.

### 3.3. Development of New TransfersomILs

In this section, new TransfersomILs (transfersomes containing ILs) were developed based on the pre-optimized formulation by incorporating the ILs into the nanodelivery systems. Rutin was loaded into these systems at the maximum amount that it is soluble in water, in each water:IL mixture, or in water:IL:IL combinations. Then, the impact of the ILs or of their combinations on the transfersomal properties was assessed.

#### 3.3.1. Physicochemical Characterization

After preparation, the physicochemical properties of all transfersomes were evaluated, namely, the D_h_, PDI, ZP, AE, and LC (Table 4). 

For the optimized formulation (containing rutin but without ILs), the obtained D_h_ was slightly smaller than that of the blank transfersomal formulation produced without rutin nor ILs. Interestingly, all transfersomes containing ILs presented a smaller size, with a D_h_ ranging between 71–83 nm. This indicates that the incorporation of ILs into the nanovesicular systems may be a worthwhile tactic, by allowing to obtain smaller systems that may facilitate skin penetration as previously discussed. Furthermore, for all the formulations, the PDI values were between 0.22 and 0.26, suggesting that the developed TransfersomILs also displayed a uniform size distribution.

Regarding zeta potential values, they ranged between −30 mV in the absence of ILs and −36 to −41 mV in the presence of ILs. These results indicate that the ILs may be present at the vesicles’ interface, contributing to the superficial charge of the particles. The observed tendency of ILs to reduce the ZP for values lower than −30 mV may favor the colloidal stability of the vesicles, avoiding particle aggregation during long-term storage [37].

Concerning the AE and LC, it is within these parameters that a higher difference was observed among the impact of ILs on the transfersomes’ properties. For instance, the optimized formulation (without IL) and the nanosystem containing only the [Emim][Br] were the formulations that presented the lowest AE and LC. These results make sense considering the lower solubility of rutin in water and in the water:[Emim][Br] mixture (Table 2), which may lead to a lower incorporation of the phenolic compound within these transfersomes. In contrast, the TransfersomILs that contain the other studied ILs ([Cho][Gly] and [Emim][Gly]) or the IL:IL combinations ([Cho][Gly]:[Emim][Br] and [Cho][Gly]:[Emim][Gly]), they all presented a higher AE and LC. This is also consistent with the higher impact in drug solubility observed for these ILs (Table 2). Nonetheless, between them, the TransfersomILs containing [Cho][Gly] and [Emim][Gly] seem to be the ones that present the most potential in terms of upgrading the physicochemical properties of the developed transfersomes, since they led not only to a higher AE and LC but also to lower values of D_h_ and ZP.

Overall, these results highlight the relevance of developing TransfersomILs, since the presence of ILs within the nanovesicular systems allows a higher drug loading, while leading to a lower particle size, which is an asset for cutaneous applications. Moreover, ILs may also promote the colloidal stability of the vesicles, counteracting one of the main disadvantages of this type of nanosystems. 

#### 3.3.2. In Vitro Release of Rutin

**Figure 3:**
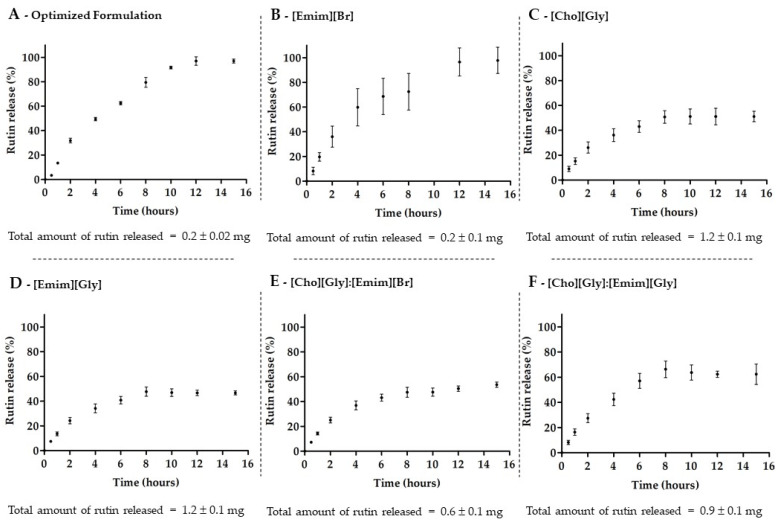
Release profile of rutin from transfersomes, in the absence of ILs (**A**), in the presence of each IL, [Emim][Br] (**B**), [Cho][Gly] (**C**), or [Emim][Gly] (**D**), or of their combinations, [Cho][Gly]:[Emim][Br] (**E**), or [Cho][Gly]:[Emim][Gly] (**F**), during 15 h in phosphate-buffered saline at pH 7.4 (mean ± SD, *n* = 3).

Following the physicochemical characterization of the formulations, the in vitro release of rutin from the transfersomes in the absence of ILs (optimized formulation) and in the presence of each IL ([Emim][Br], [Cho][Gly], or [Emim][Gly]) or IL:IL combinations ([Cho][Gly]:[Emim][Br] or [Cho][Gly]:[Emim][Gly]) was also evaluated. The release profiles and the total amount of rutin released in each case are presented in Figure 3. 

The release profiles indicate that all transfersomes, in the absence or presence of ILs, were able to release rutin in a controlled manner as previously described for rutin-loaded liposomes [62]. The results showed, once again, a very similar behavior for both the optimized formulation (without IL) and the TransfersomILs containing the [Emim][Br] alone (Figure 3A,B). Both systems led to the lowest total amount of rutin released over 15 h, even though the system allowed the release of 100% of the loaded rutin. On the other hand, the total amount of released rutin from the other TransfersomILs (Figure 3C–F) was higher compared to the optimized formulation and consistent with the solubility studies (Table 2) and the AE and LC results (Table 4). Despite the fact that the later TransfersomILs were not able to release more than 50–60% of the loaded rutin, the inclusion of [Cho][Gly], [Emim][Gly], or their combination into the nanosystem led to an increase of 5–6-fold of the total amount of rutin release in 15 h.

These results point out again the relevance of developing nanovesicular systems based on ILs and the relevance of choosing the appropriate IL or combination of ILs to be incorporated. For instance, in terms of drug release, the new TransfersomILs obtained from [Cho][Gly] or [Emim][Gly] were once more the most promising nanosystems.

#### 3.3.3. Preliminary Stability Studies

Since poor storage stability is one of the drawbacks that transfersomes may have, stability studies were also performed within the scope of this study to preliminarily assess if the ILs could alter the storage stability of the developed transfersomes. 

**Figure 4:**
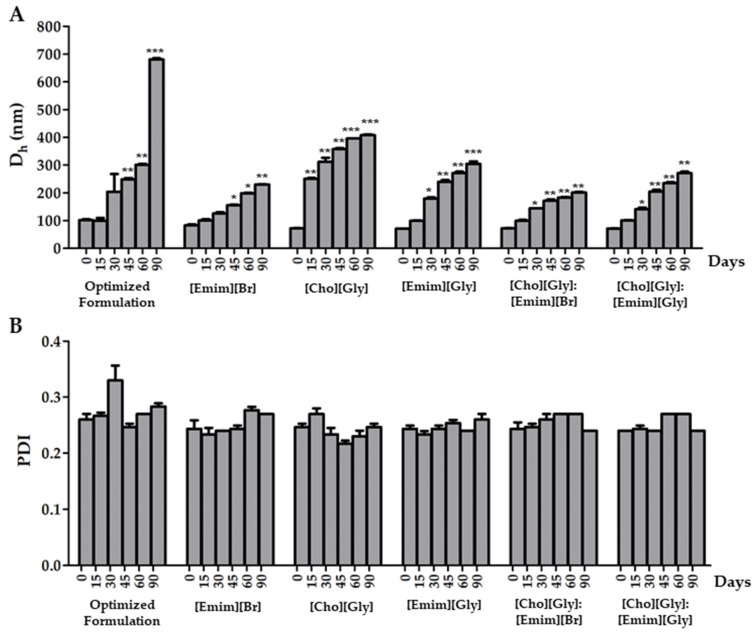
Variation of the hydrodynamic diameter (D_h_) (**A**) and the polydispersity index (PDI) (**B**) of rutin-loaded transfersomes in the absence (optimized formulation) or in the presence of each IL, or of their combinations, upon refrigerated (5 ± 2 °C) storage for 90 days. Mean ± SD, *n* = 3, * *p* < 0.05, ** *p* < 0.01, and *** *p* < 0.001.

All the prepared formulations containing rutin and in the absence (optimized formulation) or in the presence of the ILs, or of their combinations, were stored in refrigerated conditions over a period of 90 days. Then, the D_h_ and PDI of the formulations were evaluated at different time points (15, 30, 45, 60, and 90 days) to assess the impact of the ILs on both parameters, over that period (Figure 4).

When considering the vesicles’ diameter, it was possible to observe from our results (Figure 4A) that D_h_ was sensitive to the storage conditions. Indeed, an increase in D_h_ was observed for all of the formulations within the 90 days. Nonetheless, it was interesting to note that all formulations containing ILs presented a less pronounced increase in diameter over time. This result is in line with the obtained ZP data (Table 4), as the inclusion of ILs caused an increase in superficial charge, resulting in a less pronounced aggregation phenomena. Among TransfersomILs, [Cho][Gly] was the IL displaying the lowest stabilizing properties in terms of the vesicles’ size. In fact, all TransfersomILs containing an IL derived from the imidazole cation allowed the maintenance of the D_h_ below 300 nm, which is suitable for a cutaneous application [37]. Moreover, these results indicate that the presence of ILs derived from the imidazole cation may be quite relevant to improving the storage stability of the developed TransfersomILs. This may be due to the higher ability of imidazole cations to intercalate within the lipid bilayer due to enhanced lipophilic characteristics in comparison with choline cations, mainly provided by the side chain present in the imidazole cation.

In terms of PDI, the results were mostly below 0.3 and with no statistically significant differences between all of them (Figure 4B). This shows that for the developed transfersomes, the uniformity of size distribution was not affected upon storage within the studied period of time.

Bearing all the data from this study in mind, when considering the physicochemical properties (i.e., D_h_, PDI, ZP, AE, and LC), of the newly developed TransfersomILs, the formulations containing [Cho][Gly], [Emim][Gly] or the [Cho][Gly]:[Emim][Br] and [Cho][Gly]:[Emim][Gly] combinations all led to improvements when compared to the optimized formulation without IL. Nonetheless, for the IL:IL combinations, the observed improvement was less pronounced than that obtained for [Cho][Gly] or [Emim][Gly] alone. Thus, the costs associated with using two ILs may not justify using these combinations. Finally, although both formulations containing either [Cho][Gly] or [Emim][Gly] showed promising results, the preliminary stability studies seem to suggest that opting for the [Emim][Gly] may be an even superior strategy to produce upgraded transfersomes based on ILs.

The ability of ILs to upgrade skin delivery systems has been shown in other formulations such as emulsions [2,67], microemulsions [68,69], bacterial nanocellulose membranes [70], and polymeric nanoparticles [28]. In comparison with these formulations, the developed TransfersomILs may represent a further breakthrough for transdermal delivery, since (a) transfersomal systems are highly deformable and may thus stand out by presenting remarkable skin penetration abilities, and (b) the presence of ILs improve the physicochemical properties and the colloidal stability of the nanovesicular systems. 

## 4. Conclusions

In the present work, new TransfersomILs (transfersomes containing ILs) to load rutin were developed and their physicochemical properties were evaluated to ultimately assess the impact of ILs’ incorporation on the nanosystem’s performance.

The impact of the studied ILs, namely, [Emim][Br], [Cho][Gly], [Emim][Gly] and of the studied combinations [Cho][Gly]:[Emim][Br] and [Cho][Gly]:[Emim][Gly] on the cell viability of HaCaT cells and on the solubility of rutin was initially assessed. The results showed that, at the studied concentrations, all the ILs and IL:IL combinations were equally safe to be included in the developed transfersomes. In addition, apart from [Emim][Br] alone, they all led to an increase in drug solubility.

Then, the optimization of a transfersomal system was performed using a Box–Behnken factorial design (BBD). This quality-by-design approach was essential for reducing the development costs of the formulation and was found to be a robust predictive model as shown by the similarity observed between the predicted data and the corresponding obtained experimental properties. Overall, BBD proved to be a valuable strategy to develop rutin-loaded transfersomes with suitable physicochemical properties for cutaneous applications. 

Following this, the studied ILs and their combinations were incorporated into the optimized transfersomal formulation. The new TransfersomILs systems showed upgraded physicochemical properties, namely, reduced diameter and increased association efficiency, loading capacity, and total amount of rutin release. Moreover, the presence of ILs also led to an improved colloidal stability, reducing the aggregation phenomena observed during storage. It is noteworthy that, although the majority of the studied ILs improved the overall properties of the nanosystems and they all presented similar cytotoxicity, [Cho][Gly] and [Emim][Gly] were found to be the most promising materials to further enhance the vesicles’ performance. They both led to systems with smaller particle size, improved colloidal stability, and higher association efficiency and loading capacity. The only distinguishing factor seems to be their performance in the preliminary stability studies, where the [Emim][Gly] stood out. Hence, this work revealed ionic liquids as a bridge to design new and refined TransfersomILs systems.

## Figures and Tables

**Table 1:** Factors and responses considered in the Box–Behnken factorial design and the corresponding tested levels and defined desirable criteria (respectively).

**Factors**	**Levels**		
	**−1**	**0**	**1**
X_1_ = Lipid concentration	4	6	8
X_2_ = EA:lipid ratio	5:95	10:90	15:85
X_3_ = Sonication time	10	15	20
**Responses**	**Desirability**		
	**Low**	**Medium**	**High**
Y_1_ = Hydrodynamic diameter, D_h_	120	110	100
Y_2_ = Polydispersity index, PDI	0.3	0.25	0.2
Y_3_ = Association efficiency, AE	70	85	100
Y_4_ = Loading Capacity, LC	0.2	0.35	0.5

**Table 2:** Cell viability of HaCaT cells exposed to [Emim][Br], [Cho][Gly], [Emim][Gly] (0.1 or 0.2% *v*/*v*; 24 h), and [Cho][Gly]:[Emim][Br] and [Cho][Gly]:[Emim][Gly] (0.1:01% *v*/*v*, 24 h) evaluated by MTT assay (*n* = 3, mean ± SD, expressed as percentages of the non-treated control cells). Results from rutin’s solubility studies at 25 ± 2 °C in water, in water:IL mixtures, and in water:IL:IL combinations are also presented (*n* = 3, mean ± SD, ** *p* < 0.01, *** *p* < 0.001).

Solvent	Ionic Liquid (%)	Cell Viability (%)	Rutin Solubility (mg/mL)
Water	0	100.0	0.21 ± 0.05
Water:[Emim][Br]	0.1	98.7 ± 3.3	0.21 ± 0.09
	0.2	94.4 ± 4.6	0.22 ± 0.05
Water:[Cho][Gly]	0.1	99.6 ± 5.1	0.84 ± 0.04 **
	0.2	97.1 ± 5.7	1.50 ± 0.08 ***
Water:[Emim][Gly]	0.1	99.3 ± 5.3	0.99 ± 0.04 **
	0.2	93.6 ± 6.9	1.60 ± 0.06 ***
Water:[Cho][Gly]: [Emim][Br]	0.1:0.1	94.0 ± 5.8	0.79 ± 0.03 **
Water:[Cho][Gly]: [Emim][Gly]	0.1:0.1	92.0 ± 5.7	0.92 ± 0.07 **

**Table 3:** Optimum levels of the selected factors to prepare the optimized formulation and experimental (*n* = 3, mean ± SD) and theoretical values obtained for the selected responses. The 95% confidence interval (CI) obtained from the theoretical data is also presented.

Optimized Formulation	Response	Experimental Data	Theoretical Data	−95% CI	+95% CI
4:5:95:20 (X_1_:X_2_:X_3_)	D_h_	102 ± 3	107.4	95.9	118.8
	PDI	0.26 ± 0.01	0.25	0.22	0.28
	AE	86 ± 2	83.3	77.4	89.2
	LC	0.43 ± 0.01	0.43	0.39	0.46

X_1_, lipid concentration (% *w*/*v* concentration); X_2_, EA:lipid ratio (*w*/*w*); X_3_, sonication time (min); D_h_, hydrodynamic diameter (nm); PDI, polydispersity index; AE, association efficiency (%); LC, loading capacity (%).

**Table 4:** Physicochemical properties of the produced transfersomes in the absence of rutin and IL, in the presence of rutin without IL, and in the presence of rutin with the ILs alone ([Emim][Br], [Cho][Gly], or [Emim][Gly]), or with IL:IL combinations ([Cho][Gly]:[Emim][Br] or [Cho][Gly]:[Emim][Gly]).

Formulation	Rutin (mg/mL)	IL (%)	D_h_ (nm)	PDI	ZP (mV)	AE (%)	LC (%)
Water	0	0	111 ± 5	0.22 ± 0.01	-	-	-
	0.21	0	102 ± 3	0.26 ± 0.01	−31 ± 3	86.3 ± 2.1	0.43 ± 0.01
Water:[Emim][Br]	0.22	0.2	83 ± 4 *	0.24 ± 0.02	−36 ± 2	82.1 ± 5.2	0.43 ± 0.01
Water:[Cho][Gly]	1.50	0.2	73 ± 2 **	0.25 ± 0.01	−41 ± 4 *	98.1 ± 0.1 **	3.68 ± 0.01 ***
Water:[Emim][Gly]	1.60	0.2	71 ± 1 **	0.24 ± 0.01	−39 ± 5 *	98.7 ± 0.1 **	3.70 ± 0.02 ***
Water: [Cho][Gly]:[Emim][Br]	0.79	0.1:0.1	72 ± 1 **	0.24 ± 0.01	−38 ± 3 *	93.6 ± 0.2 *	2.20 ± 0.01 ***
Water: [Cho][Gly]:[Emim][Gly]	0.92	0.1:0.1	73 ± 1 **	0.24 ± 0.01	−36 ± 3	97.9 ± 0.1 **	1.76 ± 0.01 ***

IL, ionic liquid; D_h_, hydrodynamic diameter; PDI, polydispersity index; ZP, zeta potential; AE, association efficiency; LC, loading capacity. *n* = 3, mean ± SD, * *p* < 0.05, ** *p* < 0.01, and *** *p* < 0.001.

## Data Availability

The data presented in this study are available in this manuscript or in the Supplementary Materials.

## Supplementary Materials

The following are available online at https://www.mdpi.com/article/10.3390/nano12010007/s1, Table S1: Description of the rutin-loaded transfersomes produced by Box–Behnken factorial design. Both the factors used to prepare the transfersomes and the obtained responses are presented; Table S2: Results from the regression analyses performed for hydrodynamic diameter (D_h_), polydispersity index (PDI), association efficiency (AE), and loading capacity (LC) using the two-way interaction model (linear vs. quadratic) considering the effect of lipid concentration (X_1_), EA:lipid ratio (X_2_), and sonication time (X_3_).
