# Supplementary material for: TransfersomILs: From Ionic Liquids to a New Class of Nanovesicular Systems
Source: Nanomaterials (Basel). 2021 Dec 21;12(1):7. doi: 10.3390/nano12010007 (PMC8747046; doi:10.3390/nano12010007)
Supplement: Supplementary file 1 [file nanomaterials-12-00007-s001.zip › nanomaterials-1494652 supplementary.pdf]

nanomaterials

MDPI

Supplementary Material

TransfersomILs: From Ionic Liquids to a New Class of Nanovesicular Systems

Ana Júlio [1,2], João Guilherme Costa [1], Catarina Pereira-Leite [1,3,†] and Tânia Santos de Almeida [1,4,*,†]

1 CBIOS - Universidade Lusófona's Research Center for Biosciences & Health Technologies, Campo Grande 376, 1749-024 Lisboa, Portugal; ana.julio@ulusofona.pt (A.J.); jgcosta@ulusofona.pt (J.G.C.); catarina.leite@ulusofona.pt (C.P.-L.)

2 Department of Biomedical Sciences, University of Alcalá, Ctra. Madrid-Barcelona Km. 33.600, Alcalá de Henares, 28871 Madrid, Spain

3 LAQV, REQUIMTE, Departamento de Ciências Químicas, Faculdade de Farmácia, Universidade do Porto, 4050-313 Porto, Portugal

4 Centro de Química Estrutural, Faculdade de Ciências, Universidade de Lisboa, Campo Grande, 1749-016 Lisboa, Portugal

* Correspondence: tania.almeida@ulusofona.pt; Tel.: +35-12-1751-5500

† Shared senior authorship.

Table S1. Description of the rutin-loaded transfersomes produced for Box-Behnken factorial design. Both the factors used to prepare the transfersomes and the obtained responses are presented.

Sample	X_1	X_2	X_3	D_h	PDI	AE	LC
1	4	5:95	15	107 ± 1	0.25 ± 0.01	79.4 ± 1.4	0.40 ± 0.01
2	8	5:95	15	116 ± 1	0.27 ± 0.01	91.3 ± 0.4	0.23 ± 0.01
3	4	15:85	15	112 ± 1	0.27 ± 0.01	85.3 ± 0.4	0.43 ± 0.01
4	8	15:85	15	114 ± 3	0.27 ± 0.01	88.5 ± 0.9	0.22 ± 0.01
5	4	10:90	10	109 ± 1	0.27 ± 0.01	74.4 ± 0.5	0.37 ± 0.01
6	8	10:90	10	115 ± 2	0.26 ± 0.01	85.5 ± 0.4	0.21 ± 0.01
7	4	10:90	20	113 ± 1	0.28 ± 0.01	87.1 ± 0.4	0.44 ± 0.01
8	8	10:90	20	115 ± 3	0.28 ± 0.01	86.9 ± 0.8	0.23 ± 0.02
9	6	5:95	10	104 ± 2	0.26 ± 0.01	77.8 ± 1.9	0.26 ± 0.01
10	6	15:85	10	110 ± 2	0.26 ± 0.01	83.9 ± 1.0	0.28 ± 0.01
11	6	5:95	20	110 ± 1	0.26 ± 0.01	85.4 ± 0.1	0.28 ± 0.01
12	6	15:85	20	116 ± 2	0.27 ± 0.01	84.4 ± 0.9	0.28 ± 0.01
13	6	10:90	15	112 ± 1	0.27 ± 0.01	89.2 ± 0.7	0.30 ± 0.01
14	6	10:90	15	114 ± 1	0.28 ± 0.01	87.2 ± 0.5	0.29 ± 0.01
15	6	10:90	15	116 ± 3	0.28 ± 0.01	88.6 ± 0.7	0.30 ± 0.01

X_1, lipid concentration (% w/v); X_2, EA:lipid ratio (w/w); X_3, sonication time (min); D_h, hydrodynamic diameter (nm); PDI, polydispersity index; AE, association efficiency (%); LC, loading capacity (%).

Table S2. Results from the regression analyses performed for hydrodynamic diameter (D_h), polydispersity index (PDI), association efficiency (AE), and loading capacity (LC) using the two-way interaction model (linear vs quadratic) considering the effect of lipid concentration (X_1), EA:lipid ratio (X_2), and sonication time (X_3).

Factor	D_h		PDI		AE		LC	
	**Coef.**	***p***	**Coef.**	***p***	**Coef.**	***p***	**Coef.**	***p***
Intercept	**111.8**	**0.00002**	**0.27**	**0.00004**	**84.1**	**0.00001**	**0.3**	**0.00003**
X_1	5	0.079	0.005	0.365	**6.92**	**0.012**	**-0.19**	**0.0005**
X_1^2	-0.63	0.609	0.0008	0.808	0.83	0.261	**-0.03**	**0.01**
X_2	3	0.181	0.008	0.192	1.95	0.126	0.01	0.146
X_2^2	2.38	0.150	0.01	0.069	1.43	0.116	0.007	0.142
X_3	3.33	0.155	0.01	0.113	**6.05**	**0.016**	**0.03**	**0.016**
X_3^2	1.63	0.259	0.003	0.383	**4.03**	**0.017**	**0.02**	**0.04**
X_1X_2	-3.5	0.222	-0.01	0.225	**-4.45**	**0.049**	-0.02	0.074
$X_1X_2^2$	-0.75	0.649	-0.008	0.208	-1.1	0.269	0.003	0.602
$X_1^2X_2$	2.25	0.253	-0.003	0.602	0.45	0.598	$4x10^{-18}$	1
X_1X_3	-2	0.423	0.005	0.478	**-5.65**	**0.031**	**-0.03**	**0.049**
$X_1^2X_3$	2	0.293	-0.005	0.345	-1.5	0.175	-0.02	0.050
X_2X_3	0	1	0.005	0.478	-3.55	0.074	-0.01	0.225

Statistically significant effects ($p < 0.05$) are highlighted in bold.
